# Homœpathy

**Published:** 1851-09

**Authors:** 


*Homœopathy.*—"Dr. Marcy is one of the *thousand or more* physicians of the Old School who have become Homœopathists."

Thus boasts the Rev. Dr. Griswold, editor of the International Magazine, the veracious character of which may be inferred from this mercenary puff, which has thus been smuggled into its columns. There is not a whit more truth in the italicized statement of this paragraph than there is in the "professional eminence!" or "liberal fortune!" which are both ascribed to the *pictured* subject of this puff—no doubt, at his own instance; though he knew that all who know him would laugh at such ludicrous pretensions, and that none but strangers could be gulled by the imposition.

But our allusion to so disgusting a subject is not on personal, but on general grounds. Instead of "a thousand, or more" physicians, with or without "professional eminence," who in endless reiteration are claimed as having "become Homœopathists," we claim to have shown by actual statistics of the sect, at home and abroad, that their numbers are insignificant; nor can "a thousand," or a moiety of such, be named, including all the countries of the earth.

It is indeed extraordinary that a much greater number of Homœopaths have not been rallied under the banner of the tribe, since they are, in the general, better paid than any other species of the genus quack; especially when in our large cities so many regular physicians are comparatively unemployed, and often dependent and needy. And yet, strange as it may be, it is not the less true, that in New York, as in other cities—it will be found, that in America at least—no physician of "professional eminence," or even acknowledged ability, has become a Homœopath, unless there were reasons obvious enough in his proverbial want of success; or after the forfeiture of his character, either among the profession or the public, or both. Instances are not rare, here or elsewhere, which might be cited in proof.

Indeed we defy the whole sect to produce an example of a medical scholar whose professional ability was acknowledged by the fraternity, or who was in successful practice by reason of public appreciation, who has become, or is now a Homœopath. If there be one such, out of the thousand or more, let him be named and located, that we may chronicle him as a rara avis—a black swan.—*New York Medical Gazette.*

